# Willingness to commute among future physicians: a multicenter cross-sectional survey of German medical students

**DOI:** 10.1186/s12995-018-0200-2

**Published:** 2018-05-29

**Authors:** Johannes Quart, Tobias Deutsch, Solveig Carmienke, Susanne Döpfmer, Thomas Frese

**Affiliations:** 1 Department of General Practice, Medical Faculty, University of Leipzig, Leipzig, Germany; 2 Institute of General Practice and Family Medicine, Martin-Luther-University Halle-Wittenberg, Halle/Saale, Germany; 3 Institute of General Practice, Charité-Universitätsmedizin Berlin, Berlin, Germany

**Keywords:** Physician shortage, Commuting, Non-urban areas, Primary care, General practice

## Abstract

**Background:**

Many countries are faced with a decrease in physicians in non-urban areas. Especially for regions with decreasing populations, temporary solutions like commuting models might be a suitable option. So far, little is known about the willingness to commute among future physicians.

**Methods:**

In this multicenter, cross-sectional survey, five years of medical students (8th to 10th semester) from three German universities (Charité Berlin, Halle, Leipzig) were questioned about their willingness to commute to work, the maximum acceptable commute time, and how several job-related factors might enhance the attractiveness of commuting.

**Results:**

Altogether 1108 of 1203 (92.1%) students completed the questionnaire. For 55.9% of the participants it was imaginable to commute to a non-urban area in the future. The most important job-related factors that would increase the attractiveness of such a commuting model were remuneration of the commuting time, higher remuneration in general, working self-employed in a joint practice with 2–3 physicians, existence of a specifically qualified “supply assistant”, provision of a home office, good public transport connection, and a driver service. The maximum acceptable commute time was on average 39.0 min (one-way). If the way to work would be a salaried integral part of the normal working time, the participants stated they would accept traveling 51.2 min (one-way).

**Conclusions:**

Most future physicians are open-minded regarding models of commuting. The attractiveness of such models can be increased mainly through higher remuneration, reduction of the physicians’ burden, and comfortable modes of transport.

## Abstract German

**Hintergrund:**

Viele Länder stehen vor der Herausforderung eines zunehmenden Ärztemangels in ländlichen Regionen. Insbesondere für Gebiete mit rückläufigen Bevölkerungszahlen könnten Pendelmodelle eine praktikable temporäre Lösung darstellen. Über die Pendelbereitschaft zukünftiger Ärzte ist bisher wenig bekannt.

**Methoden:**

In dieser multizentrischen Querschnittsstudie wurden fünf Jahrgänge von Medizinstudierenden (8. bis 10. Semester) aus drei Universitäten (Berlin, Halle, Leipzig) zu Ihrer Pendelbereitschaft befragt. Außerdem wurden maximal akzeptable Fahrzeiten und der Einfluss verschiedener Arbeitsbedingungen auf die Attraktivität des Pendelns erhoben.

**Ergebnisse:**

Von 1203 Studierenden in den untersuchten Jahrgängen erhielten wir 1108 analysierbare Fragebögen (92.1%). Für 55.9% der Teilnehmer war es vorstellbar, zukünftig in ländliche oder kleinstädtische Regionen zu pendeln. Den stärksten positiven Einfluss auf die Attraktivität eines solchen Pendelmodells hatten eine Vergütung der Fahrzeiten, eine generell höhere Vergütung der Tätigkeit, die Arbeit in einer Gemeinschaftspraxis (2–3 Ärzte), die Entlastung durch qualifizierte „Versorgungsassistenten“, die Bereitstellung eines Home-Office, eine gute Anbindung an öffentliche Verkehrsmittel und ein Fahrservice. Grundsätzlich betrug die maximal akzeptable Fahrzeit (eine Strecke) durchschnittlich 39.0 Minuten. Wäre die Fahrzeit bezahlt und Teil der regulären Arbeitszeit, würden die Teilnehmer durchschnittlich 51.2 Minuten je Strecke akzeptieren.

**Schlussfolgerung:**

Die zukünftigen Ärzte stehen Pendelmodellen mehrheitlich offen gegenüber und könnten primär über Vergütung, Reduktion der Arbeitsbelastung und komfortable Beförderungsmöglichkeiten motiviert werden.

## Background

There is a decrease in physicians in non-urban areas in many countries, including Germany [1–3]. Particularly the growing shortage of primary care physicians will increasingly threaten adequate medical care for an aging population in many regions [4]. The perspective of living in a non-urban area seems to be relatively unattractive for young physicians today [5]. The reasons are diverse and include perceptions of demanding working conditions, inadequate payment, limited opportunities for personal and professional development, less job opportunities for spouses as well as limited educational opportunities for children [2, 6]. Regardless of the medical profession, in Germany there is a general trend towards a concentration of the population in attractive urban regions. Interestingly, although particularly shaped by young adults starting out at their first job, this trend is not primarily linked to the local availability of jobs [7].

Several studies have been conducted to identify factors attracting or inhibiting young physicians establishing or taking over rural doctors’ offices [8]. However, in some regions the population and thus the supply needs are continuously decreasing, querying the economic viability of local doctors’ offices in the long run [9].

Particularly for those regions temporary solutions like commuting models might be a pragmatic option to fill gaps in healthcare availability. So far, little is known about future physicians’ attitudes regarding commuting. This study aimed to examine medical students’ willingness to commute in the future, maximum acceptable distances, and how different job-related factors might positively influence the willingness to commute.

## Methods

### Sampling and design

Between May 2015 and May 2016 we performed a cross-sectional questionnaire-based survey among 5 years of medical students (8th to 10th semester of altogether 10 semesters plus 2 semesters final clinical year) from three German universities (Charité Berlin, Halle, and Leipzig). In Leipzig, students completed the questionnaires at the beginning of a mandatory geriatric self-experience course (10th semester) and prior to the written exam following the lecture series in general practice (8th semester), respectively. In Halle, students completed the questionnaires at the end of a mandatory two-week general practice clerkship (9th semester). In Berlin, students answered the questionnaire at the beginning of a mandatory general practice seminar (10th semester).

### Questionnaire

We used a self-developed questionnaire created by a multidisciplinary team (general practitioner, general practice resident, psychologist, and economist). To ensure comprehensibility, usability and face validity, the questionnaire was pre-tested with two medical students in advanced study years (target group). The pre-testing procedure was oriented towards the qualitative method of concurrent think aloud (CTA) and led to minor adjustments with regard to content and form.

Survey participants were questioned about relevant socio-demographic information, current career preferences, their willingness to commute to a non-urban area while living in an urban environment (as a hypothetical scenario), and the maximum acceptable commute time (one-way from home to workplace/ door to door). Furthermore, they were asked to assess how several work-related factors would positively influence their readiness to commute.

### Statistical analysis

Data were analyzed using IBM SPSS Statistics 24 for Windows. As N’s may vary due to missing values, frequencies are presented as %_valid_ (n_absolute_/n_valid_). Continuous variables are presented as mean ± standard deviation (SD). Chi-square test was used for group comparisons with regard to frequencies. Continuous variables were compared using the Mann-Whitney U test in the case of two groups and the Kruskal-Wallis test in the case of more than two groups. Statistical significance was assumed for *p* < 0.05.

### Cartographic presentation (isochrones map)

To illustrate the practical consequences of our results regarding the maximum accepted commute times within different scenarios we created a geographical map with isochrones lines. As a map of Germany would have been too complex, we decided to show the areas of Saxony potentially covered by commuting. The underlying commuting times were nevertheless derived from the whole sample (including Halle, Leipzig, and Berlin). As in Germany medical study places are awarded centrally, primarily based on school leaving examinations, students’ places of origin, study places, and later workplaces are often not the same. Considering this, although focusing on the example of Saxony, the use of all data seemed to be most realistic. We used ISO4APP API [10], a software tool based on “openstreetmap” [11], which is made available for free under the Open Database License [12]. The isochrones lines were created for the scenario of driving by car at average daily traffic from the city-center of one of the three big cities in Saxony with more than 100,000 inhabitants (Dresden, Chemnitz, and Leipzig).

## Results

Out of altogether 1203 students 1114 returned a questionnaire. After the exclusion of six only fragmentarily filled out forms the analyzable sample size was 1108, corresponding to a response rate of 92.1%. The participants’ age was on average 25.3 ± 3.2 years and 64.4% (714/1108) were women. Detailed sample characteristics are shown in Table 1. For 11.8% (126/1069) of the students, general practice was the currently preferred career option. With regard to their future place of residence 71.1% (779/1096) of the students stated that they could imagine to live in a big city, 64.6% (708/1096) could imagine to live in a small town and 42.7% (468/1096) in a rural area (multiple answers were possible). For 24.5% (269/1096) of the participants living in a big city was the only option.

**Table 1:** Sample characteristics

Variable	valid (N)^a^	N (%)^b^
Sub-Sample (university, year, course, semester)	1108	
Leipzig, 2016, geriatric self-experience course, 10th semester		259 (23.4)
Leipzig, 2015, geriatric self-experience course, 10th semester		259 (23.4)
Leipzig, 2016, general practice exam, 8th semester		274 (24.7)
Berlin, 2015, general practice seminar, 10th semester		159 (14.4)
Halle, 2015, two-week general practice clerkship, 9th semester		157 (14.2)
Age [mean ± SD]	1103	25.3 ± 2.3
Female gender	1108	714 (64.4)
Living in a stable relationship	1089	680 (62.4)
Having children	1094	98 (9.0)
At least one parent with university degree	1100	837 (76.1)
Being a physician’s child	1106	290 (26.2)
Family or friends in general practice	1104	341 (30.9)
Pre-existing concluded education in a medical occupation	1103	244 (22.1)
Mainly grew up in …	1098	
... big city		422 (38.4)
… small town		369 (33.6)
… rural area		307 (28.0)

^a^N’s vary due to missing values

^b^Unless otherwise indicated

Regarding whether they can imagine commuting to a rural/small-town area for work while living in a big city (as a hypothetical scenario), 10.4% (115/1106) of the participants answered *“definitely yes”*, 45.5% (503/1106) *“rather yes”*, 35.7% (395/1106) *“rather no”*, and 8.4% (93/1106) *“definitely no”*. Among those who considered a big city as their future place of residence 60.7% (472/777) could imagine commuting to a rural/small-town area (*“definitely yes” or “rather yes”).* Among those who stated that living in a big city is the only option 44.2% (118/267) could imagine commuting (*“definitely yes” or “rather yes”)*. Based on bivariate comparisons we found no significant associations between the willingness to commute (*“definitely/rather yes”* vs. *“rather/definitely no”*) and the variables gender, age, university, stable relationship, having children, regional background, pre-existing concluded education in a medical occupation, and current GP career preference (data not shown).

**Fig. 1:**
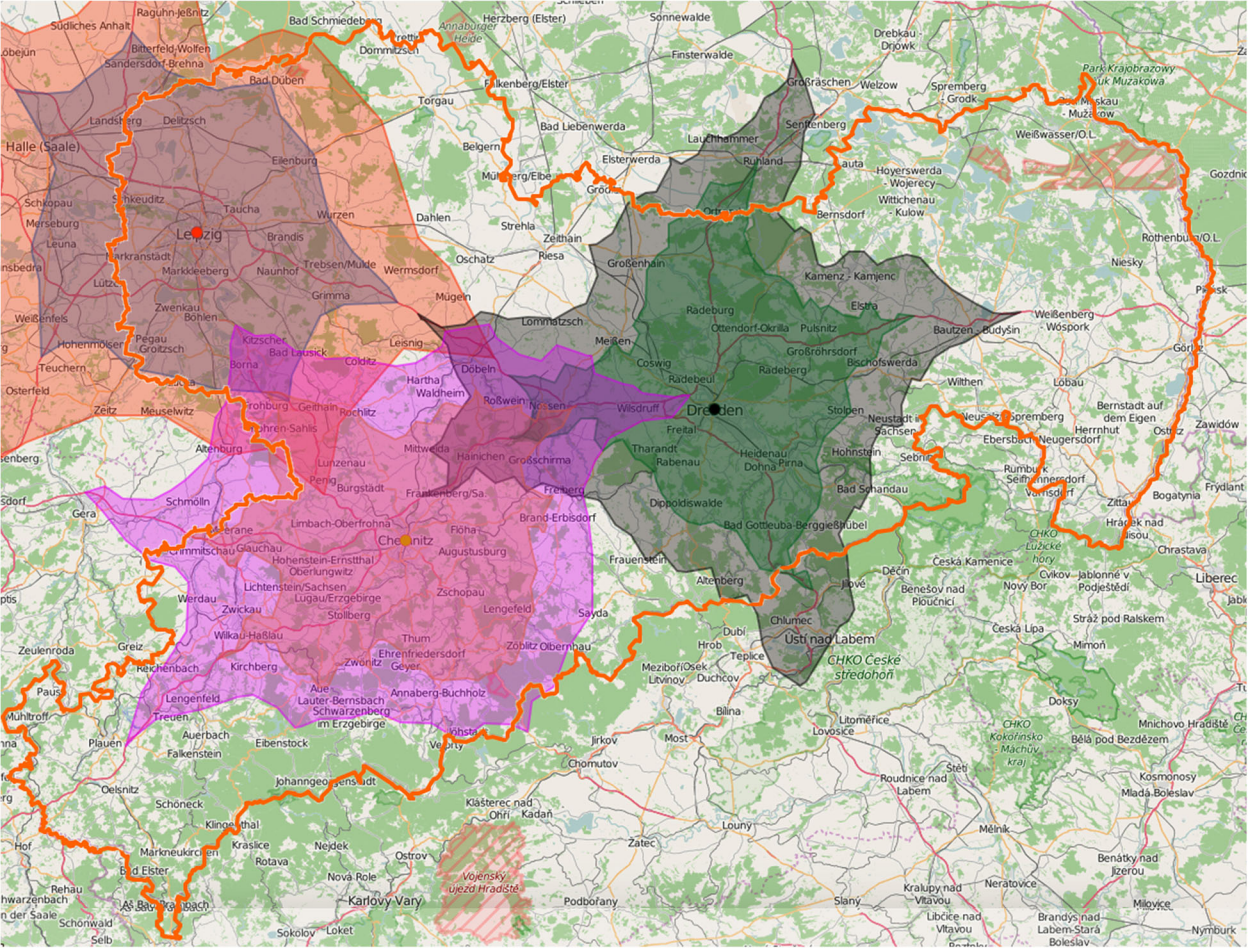
Areas potentially covered by commuting in Saxony*. * related to the centers of the cities with more than 100,000 inhabitants (Dresden, Leipzig, Chemnitz) and an accepted time getting to work of 39.0 min. Outer lines show coverage by an increased accepted commute time in case of payment for travel time and integrating it into working time (51.2 min)

Basically, the maximum acceptable commute time (one-way) for the surveyed students was on average 39.0 ± 13.3 min (*N* = 1098). Regarding the scenario of living in an urban but working in a rural/small-town environment, the maximum acceptable commute time varied depending on different modes of transport and payment (Table 2). The participants accepted the longest commute time if it would be a salaried and integral part of the working time (51.2 ± 22.6 min). Based on these time specifications (basic and longest acceptable time), an exemplary map of Saxony showing areas potentially covered by commuting is presented in Fig. 1.

**Table 2:** Maximum acceptable commute time to work (one-way) depending on different conditions

Maximum acceptable commute time (one-way) if …	valid	Mean ± SD	Quartiles (minutes)		
	N	in minutes	25%	50%	75%
… I go by car	1084	36.1 ± 12.9	30.0	30.0	45.0
… I go by public transport	1083	40.3 ± 15.2	30.0	40.0	45.0
… I can take part in a car pool	1064	35.5 ± 13.9	30.0	30.0	45.0
… I get picked up by a driver service	1065	39.1 ± 15.6	30.0	30.0	45.0
… time getting to work is paid as working time, additional to it	1075	47.0 ± 18.3	30.0	45.0	60.0
… time getting to work is paid as working time, included into it	1069	51.2 ± 22.6	30.0	50.0	60.0
… time getting to work is utilizable for organizational tasks	1066	43.9 ± 19.0	30.0	45.0	60.0

We found no significant associations between the maximum acceptable commute time (one-way) and the variables gender, age, stable relationship, having children, pre-existing concluded education in a medical occupation, and current GP career preference (data not shown). However, there were significant differences depending on university (Halle: 35.6 ± 12.1 min, Leipzig: 38.1 ± 12.6 min, Berlin: 46.6 ± 15.5 min; *p* < 0.001) and regional background (mainly grew up in big city: 40.9 ± 14.0 min, small-town: 38.8 ± 13.7 min, rural area: 36.6 ± 11.5 min; *p* < 0.001).

**Fig. 2:**
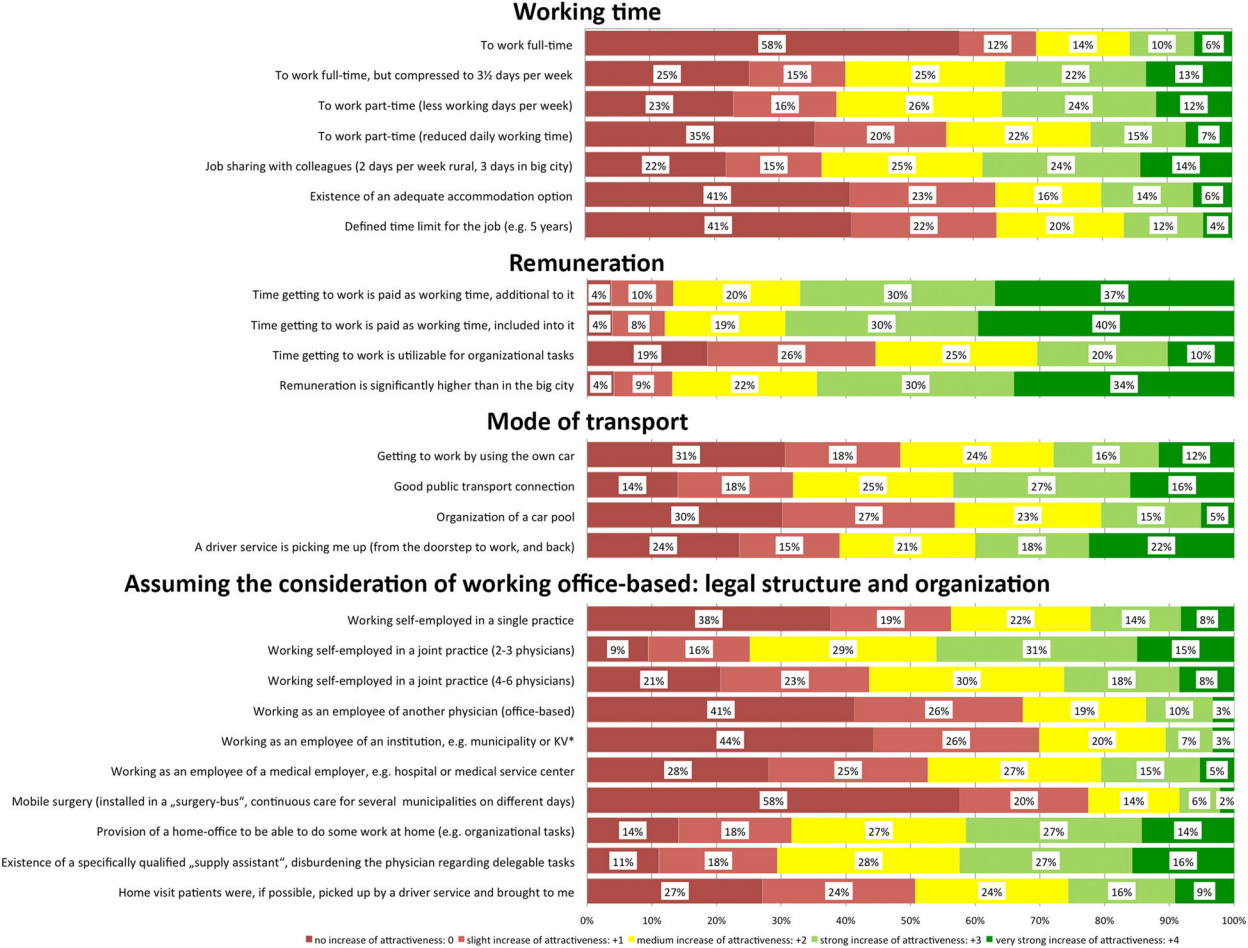
Influence of several job-related conditions on the attractiveness of commuting to a rural/small-town area (100% bar chart)

The participants were asked to what extent various job-related factors would increase the attractiveness of commuting to a rural/small-town area (scale from 0 = *‘no increase’* to 4=*‘very strong increase’*). The respective results are provided in Fig. 2 as relative frequencies (100% bar charts) and in Table 3 as means±SD (overall and depending on gender and current GP career preference).

**Table 3:** Influence of several job-related conditions on the attractiveness of commuting to a rural/small-town area – differences due to gender and general practice career preference

Potential influence factor	Students’ assessment of the increase of the attractiveness of commuting *(0 = no increase, + 1 = slight increase, + 2 = medium increase, + 3 = strong increase, + 4 = very strong increase)*						
	All	Male	Female	p*	GP career preferred	Others	p*
	Mean ± SD	Mean ± SD	Mean ± SD		Mean ± SD	Mean ± SD	
Working time							
To work full-time	0.9 ± 1.3	1.1 ± 1.4	0.8 ± 1.2	**0.003**	1.0 ± 1.3	1.0 ± 1.3	0.882
To work full-time, but compressed to 3½ days per week	1.8 ± 1.4	1.8 ± 1.4	1.8 ± 1.4	0.984	2.1 ± 1.4	1.8 ± 1.4	**0.044**
To work part-time (less working days per week)	1.9 ± 1.3	1.6 ± 1.3	2.0 ± 1.3	**< 0.001**	2.2 ± 1.3	1.8 ± 1.3	**0.005**
To work part-time (reduced daily working time)	1.4 ± 1.3	1.1 ± 1.2	1.5 ± 1.3	**< 0.001**	1.9 ± 1.4	1.3 ± 1.3	**< 0.001**
Job sharing with colleagues (2 days per week rural, 3 days in big city)	1.9 ± 1.4	1.8 ± 1.3	2.0 ± 1.3	**0.002**	2.0 ± 1.5	2.0 ± 1.3	0.954
Existence of an adequate accommodation option	1.2 ± 1.3	1.4 ± 1.3	1.2 ± 1.3	**0.012**	1.2 ± 1.3	1.2 ± 1.3	0.723
Defined time limit for the job (e.g. 5 years)	1.2 ± 1.2	1.2 ± 1.2	1.2 ± 1.2	0.915	1.1 ± 1.2	1.2 ± 1.2	0.243
Remuneration							
Time getting to work is paid as working time, additional to it	2.9 ± 1.1	2.9 ± 1.1	2.9 ± 1.1	0.876	2.9 ± 1.2	2.9 ± 1.1	0.683
Time getting to work is paid as working time, included into it	2.9 ± 1.1	2.9 ± 1.2	3.0 ± 1.1	0.266	3.0 ± 1.1	2.9 ± 1.1	0.334
Time getting to work is utilizable for organizational tasks	1.8 ± 1.3	1.9 ± 1.2	1.7 ± 1.3	**0.048**	1.9 ± 1.3	1.8 ± 1.2	0.174
Remuneration is significantly higher than in the big city	2.8 ± 1.1	2.8 ± 1.2	2.8 ± 1.1	0.647	2.9 ± 1.1	2.8 ± 1.1	0.436
Mode of transport							
Getting to work by using own car	1.6 ± 1.4	1.7 ± 1.4	1.6 ± 1.4	0.352	1.6 ± 1.4	1.6 ± 1.4	0.903
Good public transport connection	2.1 ± 1.3	2.1 ± 1.3	2.2 ± 1.3	0.223	2.2 ± 1.3	2.1 ± 1.3	0.539
Organization of a car pool	1.4 ± 1.2	1.3 ± 1.2	1.5 ± 1.2	**0.017**	1.4 ± 1.2	1.4 ± 1.2	0.611
A driver service is picking me up (from the doorstep to work, and back)	2.0 ± 1.5	1.9 ± 1.5	2.1 ± 1.5	**0.020**	1.8 ± 1.6	2.0 ± 1.5	0.149
Assuming the consideration of working office-based: legal structure and organization							
Working self-employed in a single practice	1.4 ± 1.3	1.5 ± 1.4	1.3 ± 1.3	**0.024**	1.8 ± 1.4	1.3 ± 1.3	**< 0.001**
Working self-employed in a joint practice (2–3 physicians)	2.3 ± 1.2	2.0 ± 1.2	2.4 ± 1.1	**< 0.001**	2.7 ± 1.2	2.2 ± 1.2	**< 0.001**
Working self-employed in a joint practice (4–6 physicians)	1.7 ± 1.2	1.6 ± 1.2	1.8 ± 1.2	**0.014**	1.8 ± 1.3	1.7 ± 1.2	0.337
Working as an employee of another physician (office-based)	1.1 ± 1.1	0.8 ± 1.0	1.2 ± 1.2	**< 0.001**	1.4 ± 1.3	1.0 ± 1.1	**0.002**
Working as an employee of an institution, e.g. municipality or KV^a^	1.0 ± 1.1	0.9 ± 1.1	1.1 ± 1.1	**0.003**	1.4 ± 1.3	0.9 ± 1.1	**< 0.001**
Working as an employee of a medical employer, e.g. hospital or medical service center	1.5 ± 1.2	1.2 ± 1.1	1.6 ± 1.2	**< 0.001**	1.6 ± 1.3	1.5 ± 1.2	0.432
Mobile surgery (installed in a ‘surgery-bus’, continuous care for several municipalities on different days)	0.8 ± 1.1	0.7 ± 1.0	0.8 ± 1.1	0.749	1.2 ± 1.3	0.7 ± 1.0	**< 0.001**
Provision of a home-office to be able to do some work at home (e.g. organizational tasks)	2.1 ± 1.3	1.9 ± 1.2	2.2 ± 1.3	**< 0.001**	2.4 ± 1.2	2.1 ± 1.3	**0.022**
Existence of a specifically qualified „supply assistant“, disburdening the physician regarding delegable tasks	2.2 ± 1.2	2.1 ± 1.2	2.2 ± 1.2	**0.010**	2.6 ± 1.2	2.1 ± 1.2	**< 0.001**
Home visit patients were, if possible, picked up by a driver service and brought to me	1.6 ± 1.3	1.6 ± 1.3	1.5 ± 1.3	0.245	1.6 ± 1.3	1.6 ± 1.3	0.926

^a^*KV* Kassenärztliche Vereinigung = Association of Statutory Health Insurance Physicians

* *p*-values < 0.05 are highlighted in bold

## Discussion

This study shows that more than half of German medical students in an advanced stage of undergraduate medical education can imagine a scenario of commuting to a future workplace in a non-urban area while living in a big city. The most crucial factors that might increase the attractiveness of commuting were associated with higher remuneration, reduction of the physicians’ burden (e.g. via joint practices or a qualified supply assistance), and comfortable modes of transport.

### Main findings in relation to other studies

We found no other studies directly addressing the factors influencing willingness of future physicians to commute to a workplace in a non-urban area while living in a big city. So far, most studies have focused mainly on how to convince physicians to settle down in rural areas [2, 6, 13–16]. Other studies have examined general health aspects of commuting or the commuting behavior of the whole population without consideration of specific professional subgroups [17–19]. Consequently, our results are only partially comparable to the existing literature.

More than half of our study participants were open-minded about commuting to a rural/small town area (“definitely yes” or “rather yes”). Thus, a substantial percentage of future physicians might be convinced, if not to live, then at least to work in non-urban areas if the right measures are taken. For general comparison, it can be stated that currently 60% of the German population commute (home and workplace in different municipalities) [18]. Despite several studies implying relations between the willingness to commute and sociodemographic variables like gender and age [19–21], we found no such associations in our data. However, it must be considered that we examined a homogenous age group of students in their mid-twenties, and respective associations might develop later on, when life circumstances change after graduation [19].

In our study the most important factors increasing the attractiveness of commuting were associated with remuneration, disburdening the physician, and comfortable modes of transportation. Previous studies investigating the willingness of physicians to establish a practice or to live and work in a rural area have also underlined the influence of money [6, 13, 22], flexible working times including part-time work [22–24], the possibility to work in a group practice [6, 13, 25], working as an employee [6, 16, 24], and a reduced workload by extended medical staff competencies and delegation (“supply assistant”) [3, 24].

Our results indicate slight but significant gender differences regarding the job-related conditions increasing the attractiveness of commuting. For women, a reduction of the workload through alternative working arrangements (part-time, job-sharing, joint practice, working as employee, home-office) as well as possibilities to avoid daily car driving (through pick up service or car pool) seem to be more attractive than for men. This may be seen in line with a previous study on young physicians’ decision to establish a practice that has also shown job cooperation possibilities, reduced workload and good reconciliation of work and family to be more important to female physicians [26].

Furthermore, we found that students interested in a GP career were significantly more attracted by part-time working models, single or small joint practices and a disburdening by “supply assistants” than their counterparts favoring other specialties. A current study examining the preferences of GP trainees regarding practice size and weekly working hours also found them to be attracted to work in small shared practices. However, this study found no direct preference to work less hours per week as long as salary is appropriate for the workload [27].

Among the participants of our study the acceptable commute time was on average 39 min. A survey from 2010 among German general practice residents found that 72% would accept up to 30 min, but only 13% up to 60 min one way [6]. For the whole German working population it has been shown that about 47% have a way to work of 10 to 30 min and about 22% a way of 30 to 60 min [28]. Our results indicate regional differences in the acceptable commute time depending on the university attended (Halle<Leipzig<Berlin). Considering the significantly different size of these cities it may be assumed that students in Berlin are simply used to longer commute times. This is supported by data of the German federal statistical office indicating longer commute times among people living in conurbations [28].

Since a nationwide map would have been hard to read, we chose Saxony as an example to illustrate areas potentially covered by the accepted commute times found in our study. Furthermore, a current report of the Saxon federal government provides sufficient and detailed comparative data concerning regional medical care supply problems [9]. Altogether, our map indicates that not every Saxon region would be able to cover healthcare supply needs via commuting models. Interestingly, the areas not covered are currently those with the biggest difficulties [9].

### Implications for practice and further research

To attract young physicians to commute to non-urban areas, the respective working conditions should be modified to ensure good remuneration and minimalized loss of leisure time. The focus should be particularly on tailored working models (flexible working times, part-time work, joint practices, job sharing, home office) as well as reducing the physicians’ workload (e.g. through support by specifically qualified medical staff with extended competencies). Furthermore, our results emphasize the importance of paying adequately for commute time and providing comfortable transportation options. Further research is needed to verify the possibly higher acceptance of commuting in comparison to conventional “live-and-work” models and to identify more factors that might increase willingness to commute. Additionally, it would be interesting to replicate our survey among residents and young medical specialists.

### Strengths and limitations

This study addresses a rarely studied, innovative topic of potential practical relevance regarding the future medical supply in non-urban areas. Medical undergraduates in advanced study years constitute a relevant target group – the future physicians. The sufficient sample size, the very good response rate and the inclusion of three different universities support the representativeness of the results. As a validated instrument fitting to our research questions and the target group was not available, we used a self-developed questionnaire. This might be discussed as a possible limitation. However, the thorough development by an experienced multidisciplinary team and the pre-testing of the questionnaire ensure at least face validity. Another limitation might result from the fact that we asked medical students about a topic that becomes really relevant only after graduation. It can’t be excluded that some of the participants’ perceptions might alter when life circumstances are changing. Furthermore, it should be considered that the list of conditions potentially influencing the attractiveness of commuting examined in our study is probably not exhaustive (e.g. we did not ask for participation in medical emergency services). The focus of this study was on the students’ general open-mindedness regarding models of commuting to a non-urban workplace while living in a big city. We didn’t discuss extensively how many students in our sample might decide to have their future place of residence in non-urban regions. As these scenarios cannot be considered as completely independent, this should be kept in mind when interpreting our results. Finally, it should be mentioned that, due to technical restrictions, our cartographic presentation is based on very limited presuppositions (using a car, starting at the city center, average traffic). Consequently, geographical conclusions should be drawn with respective care.

## Conclusion

Many future physicians are open-minded regarding models of commuting to non-urban areas. There are several possibilities to moderately increase the attractiveness of such models through customized working conditions, particularly with regard to remuneration, working time, a reduction of the physicians’ burden (e.g. joint practice, specifically qualified supply assistance), as well as the provision of comfortable modes of transport. Regions further from big cities (approximately more than 1 h) may not be able to close gaps in medical care supply through commuting models.
